# Effect of barberry (*Berberis vulgaris*) consumption on blood pressure, plasma lipids, and inflammation in patients with hypertension and other cardiovascular risk factors: study protocol for a randomized clinical trial

**DOI:** 10.1186/s13063-020-04918-7

**Published:** 2020-11-27

**Authors:** Hadi Emamat, Ali Zahedmehr, Sanaz Asadian, Hadith Tangestani, Javad Nasrollahzadeh

**Affiliations:** 1 Department of Clinical Nutrition & Dietetics, National Nutrition and Food Technology Research Institute, Faculty of Nutrition Sciences and Food Technology, Shahid Beheshti University of Medical Sciences, Tehran, P.O. 19395-4741 Iran; 2 Cardiovascular Intervention Research Center, Shahid Rajaei Cardiovascular, Medical & Research Center, Iran University of Medical Sciences, Tehran, Iran; 3 Department of Radiology, Shahid Rajaie Cardiovascular, Medical, and Research Center, Tehran, Iran; 4 Department of Nutrition, Persian Gulf Tropical Medicine Research Center, Bushehr University of Medical Sciences, Bushehr, Iran

**Keywords:** Barberry, *Berberis vulgaris*, Blood pressure, Lipids, Inflammation, CVD

## Abstract

**Background:**

Cardiovascular diseases (CVDs) remain the leading causes of morbidity and mortality in the world. Hypertension is an important and prevalent cardiovascular risk factor. The present study will be conducted to investigate the effect of barberry as a cardio-protective fruit on the blood pressure in patients with hypertension and other CVD risk factors. Furthermore, plasma concentrations of lipids and inflammatory biomarkers will be evaluated.

**Methods/design:**

This is an 8-week, prospective, single-blinded, parallel assigned, randomized controlled clinical trial (RCT) in which eligible men and women with hypertension and other cardiovascular risk factors will be randomized to either placebo powder (PP; containing 9 g maltodextrin, 1 g citric acid, 1 g milled sucrose and edible red color (*n* = 37)) or barberry powder (BP; containing 10 g milled dried barberry and 1 g of milled sucrose (*n* = 37)) groups. At baseline and after 8 weeks of intervention, plasma lipids and inflammatory markers, 24-h urinary nitrite/nitrate and sodium excretion, and 24-h ambulatory blood pressure monitoring (ABPM) will be measured. Anthropometric measures and dietary assessment will be performed as well. Data analysis will be done using SPSS version-21 software.

**Discussion:**

The interest in natural and functional food products has increased globally. This RCT will add to the growing literature for the potential antihypertensive, lipid-lowering, and anti-inflammatory effects of barberry in humans.

**Trial registration:**

ClinicalTrials.gov (NCT number) NCT04084847. Registered on 10 December 2019.

## Introduction

Cardiovascular diseases (CVDs) remain the leading causes of morbidity and mortality in the world, as they account for about 30% of mortality worldwide [1]. These diseases are also one of the major priorities of the Iranian healthcare system [2], and it is estimated that the burden of the disease will increase more than twice as the elderly population increases until the 2025 years [3]. Hypertension is an important and prevalent cardiovascular risk factor that imposes a great burden on the healthcare system [4]. According to the World Health Organization (WHO) reports, hypertension affects half of the adults worldwide and its prevalence is dramatically increasing in all age groups [5]. The production of nitric oxide (NO) from arginine in vascular endothelial cells is important in vasodilation, maintaining vascular tone maintenance, and regulation of blood pressure (BP). Most of the NO produced is metabolized to nitrite and nitrate (NOx) and excreted in the urine [6].

A significant number of patients with hypertension have several cardiovascular risk factors. Diabetes and dyslipidemia are among the most important risk factors known for CVDs [7] which may coexist with hypertension. Besides, inflammatory mediators are involved in many cardio-metabolic disorders. Several inflammatory biomarkers have been investigated to improve cardiovascular risk prediction and to monitor the disease process. Among the pro-inflammatory biomarkers, C-reactive protein (CRP) and interleukin-6 (IL-6) are associated with an increased risk of cardiovascular events and progression [8].

Epidemiologic studies have shown that a high intake of fruits and vegetables reduces the risk of developing cardiovascular disease [9]. This may be due to bioactive compounds found in fruits and vegetables such as polyphenols. Findings from experimental and epidemiological studies suggest that dietary intake of polyphenol-rich foods could be effective in reducing cardiovascular events. Higher intakes of fruit-based flavonoids have been associated with a lower risk of nonfatal myocardial infarction and ischemic stroke [10, 11]. The Berry family is rich in polyphenols such as procyanidins, quercetin, phenolic acids, and especially anthocyanins [12]. The anthocyanins are effective antioxidants in preventing CVD due to inhibiting inflammatory processes, reducing endothelial dysfunction and causing vasodilation [13]. Studies have shown that consumption of berry fruits such as Elderberry, Cranberry, Bilberry, Blueberry, Whortleberry, and Black Raspberry can have beneficial effects on CVDs [14]. Regarding hypertension, beneficial effects of berry fruits on blood pressure in healthy as well as hypertensive individuals have also been reported [15–18].

*Berberis vulgaris* commonly known as barberry is cultivated in Europe and West Asia, among other countries, and its fruit is used in several different forms for culinary purposes, jams, and soft drinks. Owing to its berberine and anthocyanins content, the effects of this plant have been investigated concerning cardiovascular risk factors in experimental studies [19, 20]. There has been, however, limited clinical trial related to the effect of barberry on cardio-metabolic parameters. Lazavi et al. showed that 200 ml of barberry juice significantly improved systolic and diastolic blood pressure, fasting blood sugar, and blood lipids in people with type 2 diabetes [21]. Shidfar et al. showed that a daily intake of 3 g of barberry extract for 3 months had beneficial effects on blood lipids and lipoproteins, glucose, and total antioxidant capacity in diabetic patients [22]. Recently, a meta-analysis showed that barberry consumption is a safe approach for the management of lipid parameters [23]. Nevertheless, the previous studies regarding the effects of barberry on cardiovascular risk factors, especially hypertension, had various limitations. These include the lack of precise monitoring of blood pressure by an accurate none-invasive method such as 24-h ambulatory blood pressure monitoring (ABPM) and also not measuring 24-h urine sodium excretion as an estimate of sodium intake for its confounding influence in evaluating the intervention’s effects on blood pressure. Furthermore, the mechanism by which barberry may modulate blood pressure has not been well studied, including its potential effect on nitric oxide production, which can be assessed by measuring the 24-h urine NOx level. Considering the high prevalence of CVD and hypertension as well as the limited number of clinical trials on the effect of barberry on hypertension and other cardiovascular risk factor, further clinical trials are required in this area. Therefore, the present randomized clinical trial will be conducted to investigate the effect of supplementation with barberry fruit on the blood pressure by 24-h ABPM and on 24-h urinary NOx and sodium excretion in patients with hypertension and other CVD risk factors. Furthermore, plasma concentrations of lipids, lipoproteins and inflammatory biomarkers will be evaluated as the secondary outcomes.

### Main aim

This study aims to investigate the effect of barberry consumption on systolic blood pressure (SBP), diastolic blood pressure (DBP), mean arterial blood pressure (MAP), plasma lipids, and inflammation status in patients with hypertension and other cardiovascular risk factors.

### Primary objective

To evaluate the effect of 8-week barberry consumption on variations in SBP measures in patients with hypertension and other cardiovascular risk factors.

### Secondary objectives

To compare within- and between-group variations in DBP, MAP, plasma lipid profile including total cholesterol (TC), low-density lipoprotein cholesterol (LDL-C), high-density lipoprotein cholesterol (HDL-C), triglyceride (TG), inflammatory biomarkers including plasma CRP and interleukin-6, and 24-h urinary NOx in patients with hypertension and other cardiovascular risk factors.

### Hypotheses

Daily consumption of barberry will improve SBP, DBP, and MAP in patients with hypertension and other cardiovascular risk factors.

Plasma levels of lipids, CRP, and IL-6 will be reduced by daily consumption of barberry in patients with hypertension and other cardiovascular risk factors.

Urinary NOx will be increased by daily consumption of barberry in patients with hypertension and other cardiovascular risk factors.

## Methods/design

### Design

**Fig. 1:**
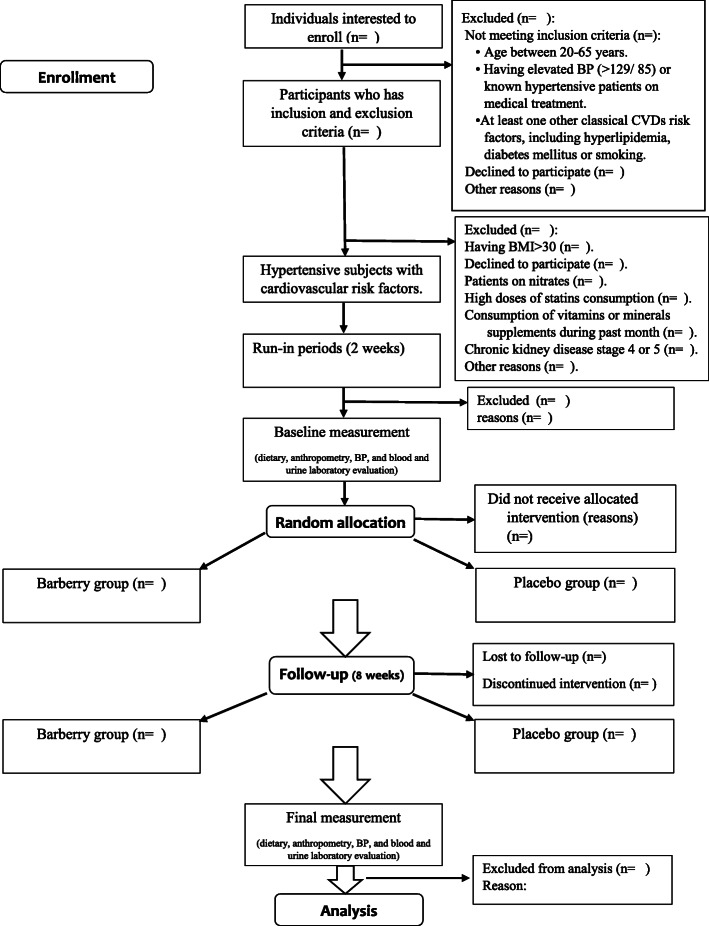
CONSORT flow diagram

This is an 8-week, prospective, single-blinded, parallel assigned, randomized controlled clinical trial (RCT) in which men and women with hypertension and other cardiovascular risk factors will be randomized to an interventional (barberry) or placebo group (Fig. 1).

### Ethics, consent, and permissions

National Nutrition and Food Technology Research Institute (NNFTRI), Shahid Beheshti University of Medical Sciences, will provide financial support for the current project. The trial will be conducted in compliance with the Declaration of Helsinki. The trial has received ethical approval from the Ethics Committee of NNFTRI (2018-10-20: Ethical code: IR.SBMU.nnftri.Rec.1397.248).

### Participant recruitment

In this clinical trial, we will recruit a total of 80 participants both men and women (each group 40) with hypertension and other cardiovascular risk factors (diabetes and/or hyperlipidemia and/or smoking) in Tehran, the capital of Iran. Volunteers among patients with a previous history of hypertension who have a medical record in the academic hospital (Rajaei Cardiovascular, Medical & Research Center, Tehran, Iran) and are regularly visited in the hospital clinics every 4–6 months if they meet the eligibility criteria will be recruited. Besides, by placing an advertisement in the hospital, other volunteer people who are referred to the hospital if they meet the eligibility criteria are also included in the study. To guarantee achieving adequate participant enrolment, the clinic staff at the hospital will be informed about the study, and informational conversation with patients will be held.

The trial is due to last 9 months. This assumes 5 months for participant recruitment and intervention, 2 months for laboratory testing, 1 month for data preparation and analysis by SPSS software, and 1 month to write the study report. The study protocol has been reported in accordance with the Standard Protocol Items: Recommendations for Clinical Interventional Trials (SPIRIT) guidelines [24] (Additional file 1, SPIRIT Checklist). The trial schedule is shown in Table 1. Informed consent will be obtained from all subjects before collecting any information. After reviewing the consent forms and answering any probable questions by study staff, those who are interested will be asked to sign the forms. A copy of the consent form will be given to participants and all original signed consent forms will be kept by the study staff (Additional file 2, consent form).

**Table 1:**
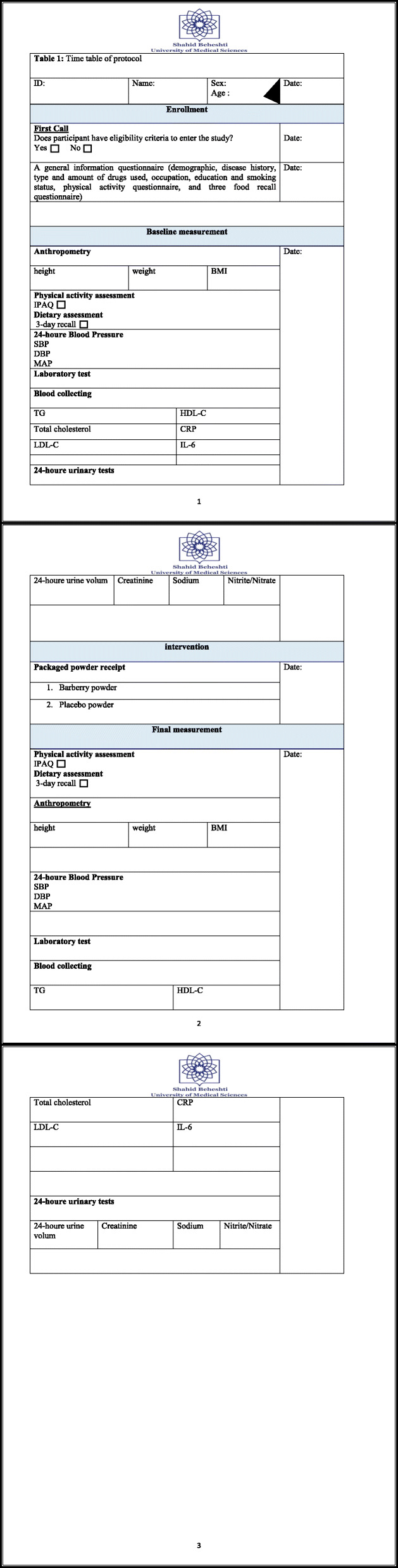
Time table of protocol

### Eligibility

The inclusion criteria for subjects include (1) willingness to participate in the study, (2) age between 20 and 65 years, (3) known hypertensive patients on medical treatment, and (4) at least one other classical CVD risk factors, including hyperlipidemia, diabetes mellitus, or smoking. Exclusion criteria include (1) unwillingness to continue participation, (2) having BMI > 30, (3) patients on nitrate drugs, (4) taking high doses of statins (Atorvastatin> 40 mg/day or Rosuvastatin> 20 mg/day), (5) consumption of vitamins or minerals supplements during the past month, and (6) having chronic kidney disease stage 4 or 5. The drop out criteria include any side effects, refusing to continue the study, missing telephone responses, and any restrictive illness. Participants are allowed to withdraw from the study at any time.

### Setting

For those who are interested in participating in the trial, cardiologist and study staff will confirm if they have eligibility criteria, and the appointment will be set for their first visit. In the first visit, the potential participants will have the proposals of the research project fully explained to them, and all participants will be asked to fill in the consent form. A general information questionnaire, including demographic information, disease history, the type and dosage of their medications, occupation, education and the smoking status, physical activity questionnaire, and three food recall questionnaire will be completed from each participant. The next visit will be after 2 weeks run-in period of weight maintenance, when baseline data including body weight and fasting blood sample will be obtained and the patient will be instructed to collect urine within the next 24 h and an ABPM device will be installed. After 24 h, the collected urine sample will be delivered and the results of the ABPM device will be stored. The patient will be given barberry or placebo packages and instruction on how to record their consumption. The next meeting will be at the end of the study to collect end-of-study data.

### Sample size

Sample size calculations were carried out using G*Power V.3.1.9.2 software [25]. The sample size was calculated based on data from Tjelle et al. [15], a relatively similar polyphenol-rich berry juice intervention study that observed a reduction in SBP of 6.2 mmHg compared to placebo. Assuming equal standard deviation in both group and by considering pooled value of the standard deviation = 8.8, a significance level of *α* ≤ 0.05 and statistical power of 80%, the minimum sample size will be 66 patients will be required to detect a change of this size in the SBP level. To account for a drop-out rate of approximately 20%, we plan to recruit 80 participants (40 randomized to each group).

### Run-in

Hypertensive patients who have other cardiovascular risk factors will be entered into the 2-week maintenance weight program after the above steps have been taken. Participants will be asked not to change their lifestyle and keep researchers informed of any changes including diet, physical activity, and medication use. At the end of the first visit, a paper sheet will be given to the individuals, containing the description of the study, a date for the next visit, and the contact number. The presence of this run-in period is essential for maintaining their weight, as well as to test the participants’ motivation to continue the intervention.

### Randomization/blinding

Participants will be randomized to receive either the barberry or the placebo product. The randomization will be performed with the aid of a sequence generated by using a random number table. For this purpose, each row of the table was considered as a block, and from the left, in the order in the table, odd numbers were considered as group A and even numbers were considered as group B. If the first two allocations were for one of the A or B, the next two allocations were considered for the second group. The sequence of each block was placed in a sealed opaque envelope. Diabetes and hyperlipidemia are two other major CVD risk factors that may coexist with hypertension. To achieve a balanced distribution of these risk factors in each of the study groups, stratified block randomization will be performed, with four strata: with a history of diabetes, without a history of diabetes, receiving statins, and not receiving statins. A block size of 4 within each stratum will be used, with a 1:1 randomization between the two groups.

This trial has a single-blinded manner. Patients are blinded to the type of product they are consuming, but the main investigator who assigns patients to intervention or placebo groups is not blinded. Placebo and barberry powdered products are given to patients in undetectable non-transparent packaging.

### Study intervention and implementation

The package of powdered products will be either a placebo powder. The barberry or placebo powders will be packaged in undetectable non-transparent wrappers. Placebo powder contains 9 g maltodextrin, 1 g citric acid, 1 g milled sucrose, and edible red color, and the barberry powder contains 10 g milled dried barberry and 1 g of milled sucrose. All dried barberry is purchased from the local market. Each patient receives 60 packages, each containing barberry or placebo powder for daily use. The daily amount of 10 g of barberry powder was selected based on a previous study by Shidfar et al. in which the amount of extract obtained from 10 g of barberry powder was investigated [22]. The time of consumption of powders can be during any time of the day and participants will not be imposed to take the supplements at a specific time of the day. Since a change in dietary habits or physical activity can affect the outcome of the study, participants will be asked not to change their daily diet and to continue their daily physical activity. Furthermore, patients will be instructed to maintain their current type/dose of oral medication during this trial period.

### Compliance

All participants will be called biweekly to assess their compliance. Participants will be asked to mark a reminder note for the daily consumption of the packages. They will be given a sheet of paper that includes the code, the next visit date, two columns entitled “one package daily” and another column entitled “Date” to record their regular consumption of package. Patients are asked to bring empty packages to the hospital at the end of the study period.

### Outcome measurements

#### Dietary assessment

Before and after the 8-week intervention, a 3-day 24-h dietary recall questionnaire will be obtained to evaluate the usual dietary intake. It includes two workdays and one weekend. Then, the obtained data from the questionnaire will be assessed using N4 software (NUTRITIONIST 4, First Data Bank, San Bruno, CA, USA).

#### Anthropometry measures

The height will be measured without shoes by a stadiometer (Seca, Germany) with a sensitivity of 0.1 cm and weight with a digital scale (808 Seca, Germany) while wearing light clothes (with no coat or raincoat) with a sensitivity of 0.1 kg before and after of 8-week intervention. Body mass index (BMI) will be calculated by dividing the weight (in kilograms) by height squared (in meters).

#### Blood pressure

Before and after of 8-week intervention, 24-h ABPM with an automatic monitor in oscillatory mode will be performed in the electrophysiology department of the hospital. The change in blood pressure during the 24-h period is recorded by this device. The blood pressure cuff is placed and fixed around the upper left arm. The digital blood pressure monitoring machine itself is attached to a belt around the waist or hangs on the neck. Technician checks that battery life is sufficient for 24 h and checks if the machine operates well. The cuff inflates every 30 min in the day and every 1 h in night time as per the settings and then slowly deflates to record the blood pressure in its memory. Patients will be asked to have their usual lifestyle and activities at this 24-h ambulatory blood pressure monitoring.

#### Laboratory investigations

##### Blood sampling

A 10-mL venous blood sample will be taken in a 12-h fasting state between 8:30 and 10:00 a.m. by the hospital lab technician and stored in a heparinized tube. Plasma separation will then be performed by centrifugation at a speed of 1000*g* for 15 min. Then, the plasma will be collected into separate micro-tubes and will be stored in a freezer at − 80 °C until laboratory analysis.

##### Blood chemistry

TG, TC, HDL-C, and LDL-C concentrations will be measured by auto-analyzer Selectra ProXL (Vital Scientific, Spankeren, The Netherlands) using commercially available kits through enzymatic colorimetric methods as per the manufacturer’s instructions. Plasma CRP will be measured using a turbidometric immunoassay kit by an auto-analyzer. Plasma IL-6 will be measured using a commercially available ELISA kit (BioLegend, USA).

##### Urinary test

Twenty-four-hour urine will be collected to measure its volume, creatinine, sodium, and nitrite/nitrate-concentrations. Urinary sodium will be measured by flame photometry to estimate sodium intake. Urine nitrite/nitrate concentrations will be determined using a colorimetric assay (Cayman Inc., USA). At baseline and the end of the study, patients are given a 24-h urine container to be filled by collected urine of the patient. The 24-h urine collection will be performed concurrently with 24-h blood pressure monitoring and the patient will return the container to the hospital the next day. The volume of collected urine will be recorded and a sample will be aliquoted into the micro-tubes to be stored in the freezer at − 80 °C until further measurements.

### Atherosclerotic cardiovascular disease risk score

Ten-year risk of atherosclerotic cardiovascular disease [26] will be determined at baseline and after the intervention.

#### Safety procedures

We will record and report all adverse events. Participants with abnormal research samples will be referred to a specialist.

#### Data collection

Data from questionnaires will be collected by HE and one other trained questioners. The collected data will be reviewed by HE at the end of the day, and in the case of any discrepancies in data collection, the questioner is asked to be more precise in the process of completing the questionnaires to reduce bias.

#### Data management and monitoring

Participants will be given a code number between 1 and 80. The master randomization list will be safely stored. Interim analyses will not be performed. When the intervention period is finished, the collected data will be entered in the SPSS software (SPSS Inc., Chicago, IL, USA).

The trial will be monitored by internal monitoring. The study leader provides monitoring of overall trial progress. During the study, regular meetings of the principal investigator and study leader will be held to discuss concerns and review the progress. Any information that is available on serious adverse events believed to be due to treatment will be supplied to the study cardiologist. If there will be suspicion of harm, discontinuation of the trial will be reviewed.

### Protocol amendments

During the study, any changes and amendments will be reviewed by the principal investigator and should be agreed to by the other study investigators. If approved, the amendments will be recorded with a justification and will eventually be reported.

#### Statistical analyses

Data analysis will be done using SPSS version-21 software. A per-protocol analysis will be carried out, in which only participants who complete the full study protocol will be included. All results will be assessed for normality, and skewed distributions will be log-transformed before analysis. To describe the quantitative data, the mean and standard deviations will be used, and to describe the qualitative data, frequency and percent will be used. Between-group differences will be tested by independent sample *t* test and Mann-Whitney *U* test as the parametric and nonparametric tests respectively. Paired-sample *t* test and nonparametric Wilcoxon signed ranks test will be used for evaluating within-group differences in normally and non-normally distributed data, respectively. Chi-square or Fisher exact tests will be used for qualitative variables. Parametric or nonparametric analysis of covariance (ANCOVA) will be used for adjusting baseline levels for comparing between groups results. In this study, *P* value less than 0.05 will be considered statistically significant.

### Auditing

The study will be conducted in accordance with the current approved protocol and National Nutrition and Food Technology Research Institute relevant regulations. An auditor from the research committee members of the National Nutrition and Food Technology Research Institute will conduct audits during and after the study.

### Ancillary and post-trial care

In these patients, if no specific side effects are observed, if desired, the barberry powder for regular use will be recommended to patients.

## Discussion

Barberry may be useful as a functional food product for the management of hypertension, hyperlipidemia, and chronic inflammation in humans, to help prevent CVD. According to a recent review, barberry has been claimed to have potential cardioprotective effects [27].. Basic animal studies have been focused on the extract forms of barberry. Hemmati et al. conducted a study on the anti-atherogenic effect of hydro-alcoholic and aqueous extract of barberry, saffron, and jujube extracts on diabetic rats. The intervention of 25 and 100 mg/kg body weight for 21 days significantly reduced serum levels of fasting glucose, triglycerides, VLDL, lipoprotein (a) with an increase in total antioxidant capacity (TAC) ,and serum adiponectin levels [28]. In the study of Fatehi et al. on hypertensive rats, the MAP was significantly decreased after 5 weeks of aqueous extract of barberry [29]. In another study, Changizi et al. showed that alcoholic extract of barberry can also improve lipid profile in a model of a high-fat diet in the rat [30].

Human studies to date have used barberry in the form of juice [21, 31] or extract [22, 32]. Daily consumption of 200 ml barberry juice for 8 weeks significantly reduced SBP and DBP, fasting blood sugar, TC, and TG in diabetic subjects [21]. In another study, 6-week supplementation with capsules containing barberry was able to reduce oxidative status in patients with metabolic syndrome [31]. In patients with diabetes, daily supplementation with barberry extract (extracted from 10 g barberry) for 3 months reduced blood lipids, glucose, and insulin and increased TAC [22]. In another clinical trial supplementation with barberry extract for 3 months reduced serum liver enzymes in patients with non-alcoholic fatty liver [32].

### Strengths

This trial will be the first to examine the effects of supplementation with dried powdered barberry on blood pressure using the 24-h ambulatory blood pressure monitoring methods. It will also be the first to investigate the effects of barberry on 24-h urinary NOx and sodium. There are also several features of this study designed to add to the existing literature regarding the effect of barberry on fasting blood lipids and inflammatory markers.

### Limitations

The study participants are comprised of hypertensive patients with other cardiovascular risk factors on medication. Therefore, a potential limitation of the study will be related to the generalizability of the results to the whole population. However, a large percentage of adults at risk for CVD are using at least one prescription medication to control hypertension or other cardiovascular risk factors. As such, although a placebo control will be applied in our study, we can assess only the complementary effect of barberry, because medications are a standard of care treatment for patients with cardiovascular risk factors. Another limitation of the study is the lack of measurement of urinary phenolic compound. Measurement of urinary flavonoid or polyphenol will undoubtedly provide invaluable information regarding the intake and absorption of polyphenols [33].

The interest in natural and functional food products has increased globally. Reliable evidence is needed to confirm the health claims related to functional foods that are obtainable through replicated, randomized, placebo-controlled, intervention trials in human subjects. In this regard, it will be interesting to conduct research aimed at evaluating the effectiveness of dried barberry which is a rich source of polyphenol for use in health promotion and disease prevention and management. This RCT will add to the growing literature for the potential antihypertensive, lipid-lowering, and anti-inflammatory effects of barberry in humans.

### Trial status

The current protocol is version 1, dated 26 March 2020, and any changes to the protocol will be communicated to all relevant parties, including participants. The recruitment process has been started since 2020-01-20 and is anticipated to last for 5 months.

## Supplementary information

**Additional file 1.** SPIRIT 2013 Checklist: Recommended items to address in a clinical trial protocol and related documents.

**Additional file 2.** Informed consent.

## Data Availability

The datasets generated and/or analyzed during the current study will be available from the corresponding author on reasonable request.

## Abbreviations

ABPM: Ambulatory blood pressure monitoring
ANCOVA: Analysis of covariance
BP: Barberry powder
BMI: Body mass index
CVD: Cardiovascular disease
CRP: C-reactive protein
DBP: Diastolic blood pressure
HDL-C: High-density lipoprotein cholesterol
IL-6: Interleukin-6
LDL-C: Low-density lipoprotein cholesterol
MAP: Mean arterial blood pressure
NO: Nitric oxide
NOx: Nitrite and nitrate
PP: Placebo powder
RCT: Randomized controlled clinical trial
SBP: Systolic blood pressure
TG: Triglyceride
TC: Total cholesterol
WHO: World Health Organization
