# Supplementary material for: Effect of barberry (Berberis vulgaris) consumption on blood pressure, plasma lipids, and inflammation in patients with hypertension and other cardiovascular risk factors: study protocol for a randomized clinical trial
Source: Trials. 2020 Nov 27;21:986. doi: 10.1186/s13063-020-04918-7 (PMC7694417; doi:10.1186/s13063-020-04918-7)
Supplement: Supplementary file 2 — Additional file 2. Informed consent. [file 13063_2020_4918_MOESM2_ESM.docx]

**
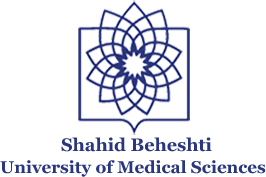
**

**Informed consent**

Mr / Ms….
We invite you to participate in our research. Information about this research is provided in this sheet and you are free to participate in this research.

You do not have to make an immediate decision. You can ask your questions from the research team to make a good decision, and can consult with anyone you want. Before signing this consent, make sure you understand all the information in this form and answer all your questions.

**Research title:** Effect of barberry (Berberis Vulgaris) consumption on blood pressure, plasma lipids, and inflammation in patients with hypertension and other cardiovascular risk factors

- I know that the main aim of this study is to investigate the effect of barberry consumption on blood pressure, and some blood markers related to cardiovascular disea such as plasma lipids
- I know my company is completely voluntary in this research and I do not have to participate in this research. I was assured that if I was not willing to participate in this research, I would not be deprived of routine diagnostic and therapeutic care and my connection with the center and physicians do not get bored.
- I know that even after agreeing to participate in the research, I can leave the research whenever I want, albeit after informing the researchers. Moreover, my abundant from the research will not deprive me of the usual services for me.
- My cooperation in this research is as follows:

I know that if I have the inclusion criteria of the research (age between 20-65 years, elevated blood pressure or or previous history of hypertension, hyperlipidemia, diabetes mellitus, BMI< 30, not using nitroglycerin or similar drug, lack of regular consumption of vitamins or minerals supplements during past month, lack of elevated serum creatinine) I can participate in this study so I respect honesty in answering questions. Then, for two weeks, I have to follow the researchers recommendations, including avoiding any weight loss diets and severe physical activity. After 2 weeks I will undergo the research. Researchers will take me 10 ml of blood to measure plasma lipid profile and inflammatory markers and I will collect my 24-houre urine, and etc. I know that I need to be at least 12 hours fasting for blood sampling. My blood pressure will also be measured using a digital blood pressure monitoring machine placed on my arm for 24-hour. Questions about my daily diet, physical activity and socioeconomic status will be completed for me. Anthropometric measurements will also be performed. It at least takes me one hour to answer these questions. I know that the study consists of two groups. People in group 1 will receive 10 grams of powdered dried barberry each day; individuals in group 2 will receive 10 grams of placebo powder per day. I will be randomly be put in one two groups of research. My assignment in each of the two groups will be completely randomized, and I cannot be involved in determining the research groups. At the end of the study, if it turned out that I was in the placebo group, I will also be given dried barberry packages for consumption if I wish so. I know that the duration of this study is 2 months. All tests include blood and urine sample, anthropometric measurements, and blood pressure, as in the beginning of the study done, are repeated at the end of the study. So, I know that 10 ml of blood, which requires at least 12 hours of fasting will be taken (2 times) at the beginning and end of the research. Researchers pledge me to compensate my travel expenses to the clinic by free report of the test results.

- The potential benefits of my participation in this study are as follows:
  The probable benefits of my participation in this study is that diagnostic tests, such as blood pressure measurements, blood lipids, etc., will be done for me two times, which is reported to me free of charge. By participating in this project, I can also receive free advice and strategies for weight management after the end of the project.
- The damages and possible complications of this study are as follows:
  The injuries and possible side effects of the participation in this study are as follows: This study does not cause any harm to me and does not have any side effects. Also, the amount of blood sample taken does not hurt me. Involvement in this research not only does not hurt me, it can be useful in diagnosing and treating my cardiovascular risk factors.
- If I do not want to participate in the research, I will provide the usual services (therapeutic, diagnostic, etc.), I will be presented with the usual method of treatment, and my routine treatment will continue.
- I know that the researchers of this study kept all my information confidential and are only allowed to publish the general results of this research without mentioning my name and profile.
- I know that the Ethics Committee of Research to monitor my rights can access my information.
- I know that this research costs me nothing. My participation is totally voluntary in this study.
- I know that participation in this research does not cost me. By participating in this research, some tests, such as blood pressure measurement, blood lipids, Blood inflammatory markers, anthropometric measurements (height, weight, etc.) 2 times (at the beginning and end of the study) are done for me free of charge. Also, during the study, I often receive free nutrition assessment. Also, all tests will be free and will not have any side effects.
- Dr. Javad Nasrollahzadeh and Hadi Emamat are introduced to me to answer my questions and I can ask for guidance whenever a problem or question related to the participation in the research came about.
  The following address, email and telephone number were provided to me as follows:
  Department of Clinical Nutrition and Dietetics, Faculty of Nutrition Sciences and Food Technology, National Nutrition and Food Technology Research Institute, Shahid Beheshti University of Medical Sciences, P.O. 19395-4741; Tehran, Iran.
- Email: [jnasrollahzadeh@gmail.com](mailto:jnasrollahzadeh@gmail.com)
- Tele: 021-22360656
  Mobile: 09122446911
- I know that if any physical and mental problems arose during and after the research because of my participation in this research, it will be the responsibility of the practitioner to treat its complications and the related damages.
- I know if I have a problem to participate or in the research process, I can contact the Ethics Committee of Shahid Beheshti University of Medical Sciences.
- This information form and informed consent will be provided in two copies and after the signature, a copy will be available to me and another copy to the researchers.

**I read and understood the above, and based on that, I declare my informed consent to participate in this research.**Participant Sign

**Dr. Javad Nasrollahzadeh as responsible researcher in this investigation is committed to do all the items mentioned above and provided the safety of all participants.**

Administer sign
